# The improvement of the quality of medical reviews of patients in seclusion in Rampton Hospital

**DOI:** 10.1192/bjo.2021.874

**Published:** 2021-06-18

**Authors:** Emma McPhail, Ian Yanson

**Affiliations:** Rampton Hospital

## Abstract

**Aims:**

Improve and standardise the quality of medical seclusion reviews (MSRs).

Acknowledge existing good practise.

Highlight areas for improvement.

Improve the awareness of doctors performing MSRs of the requirements in the Mental Health Act Code of Practice (MHA CoP)

**Background:**

MSRs are an essential clinical tool to ensure safe and consistent patient care. Patients detained in seclusion can be at heightened risk of poor mental and physical health, in addition to being a risk to themselves and others. There is clear guidance in the MHA CoP regarding what areas require to be covered in a MSR.

**Method:**

A retrospective audit of all MSRs in September 2019 across all patients within all directorates within Rampton Hospital was undertaken. 281 inpatients were identified within Rampton Hospital, and 61 of these patients were found to have had seclusion in September 2019. A total of 439 MSRs were identified for these patients.

The standard applied was the MHA CoP guidance for MSRs:

1. MSRs should be conducted in person, and should include:
2. Review of physical health
3. Review of psychiatric health
4. Assessment of the adverse effects of medication
5. Review of observations required
6. Reassessment of medication prescribed
7. Assessment of the patient's risk to others
8. Assessment of the patient's risk of self-harm
9. Assessment of the need for continuing seclusion

100% compliance with targets or a reason why it was not possible was expected to be documented.

**Result:**

The results show there is a large variation in compliance with the MHA CoP. The area with the highest compliance was the completion of reviews in person-(99.3%). The criterion with the average worst compliance was whether the need for physical observations was reviewed-(4.3%). Physical health was reviewed in 86.1% of cases, in contrast to psychiatric health at 38.3%. The adverse effects of medication and reassessment of medication prescribed were recorded in only 8.9%. The risk from the patient to others was recorded in 25.3%, whereas risk to self was recorded in 10.7%. The need for continuing seclusion was recorded in 72.7%.

**Conclusion:**

The quality of MSRs at Rampton Hospital is currently inadequate. Improvement in practice is required to meet accepted standards and ensure safe, consistent patient care. Ways to improve this are being considered, including improving the knowledge of the MHA CoP and providing a MSR template.

